# Diffuse Facial Melasma and Lentigines Treated With the NewMelan Multimodal Depigmentation Protocol: A Case Report

**DOI:** 10.7759/cureus.112167

**Published:** 2026-07-06

**Authors:** Eduardo Krulig

**Affiliations:** 1 Plastic Surgery and Aesthetic Medicine, Krulig International, Inc., Pembroke Pines, USA

**Keywords:** cosmetic dermatology, melanogenesis, photoaging, skin hyperpigmentation, skin pigmentation disorders

## Abstract

This case report describes a 75-year-old woman presenting with diffuse facial melasma, lentigines, and associated dyschromia. The NewMelan multimodal depigmentation protocol, developed by Dr. Eduardo Krulig, was utilized in the management of this patient, who was treated under his supervision.

Clinical examination demonstrated bilateral facial hyperpigmentation with uneven skin tone and photoaging-related pigmentary changes consistent with mixed melasma and lentiginous pigmentation. A physician-supervised depigmentation regimen was initiated. During the first week, the depigmenting product was applied three times daily, followed by twice daily applications during the second week. After 15 days, the protocol continued with once daily application as part of the maintenance regimen.

Marked clinical improvement in facial hyperpigmentation and skin tone uniformity was observed within approximately two weeks of treatment initiation. Serial clinical photography demonstrated substantial reduction in pigmentation intensity, near-complete resolution of the most prominent melasma lesions, and improvement in overall facial appearance. Post-treatment, the patient was instructed to continue daily maintenance therapy.

This case demonstrates short-term clinical improvement following treatment with the NewMelan multimodal depigmentation protocol. Further studies with larger patient populations and longer follow-up periods are necessary to evaluate long-term outcomes, recurrence rates, and broader clinical applicability.

## Introduction

Diffuse melasma and lentigines are common pigmentary disorders that present therapeutic challenges due to their recurrent nature and variable response to treatment [1-4]. These conditions are influenced by multiple factors, including ultraviolet and visible light exposure, hormonal changes, and chronic photodamage [5,6]. Effective management often requires a combination of topical depigmenting agents and ongoing maintenance therapy to achieve and sustain clinical improvement [7,8].

Depigmentation protocols targeting melanogenesis pathways and epidermal turnover have demonstrated short-term improvements in pigmentation and skin tone [9,10]. The NewMelan multimodal depigmentation protocol is designed to reduce melanin synthesis, accelerate epidermal renewal, and improve pigmentation uniformity [11,12].

This case report describes a 75-year-old woman with diffuse facial melasma and lentigines who underwent treatment with the NewMelan multimodal depigmentation protocol, highlighting the observed clinical improvements and short-term efficacy of this approach [13,14].

## Case presentation

The patient is a 75-year-old woman presenting with diffuse facial hyperpigmentation and multiple lentigines, predominantly affecting the malar regions and forehead. She reported gradual darkening of the skin over several years, with exacerbation after sun exposure. Her medical history was unremarkable, and she had no prior systemic treatments for pigmentary disorders. The patient provided written informed consent for treatment and publication of clinical images.

On examination, there was bilateral facial hyperpigmentation with uneven skin tone, consistent with mixed melasma and lentiginous pigmentation. No inflammation, erythema, or secondary lesions were observed. Dermoscopic evaluation was not performed.

A structured depigmentation regimen using the NewMelan multimodal protocol was initiated. The topical depigmenting formulation contained arbutin, 1.5%; kojic acid, 1.0%; and azelaic acid, 1.5%. These active ingredients were selected because of their complementary depigmenting properties and were incorporated to target multiple pathways involved in melanogenesis. The application schedule consisted of three times daily during the first week, twice daily during the second week, and once daily thereafter as maintenance therapy.

**Figure 1:**
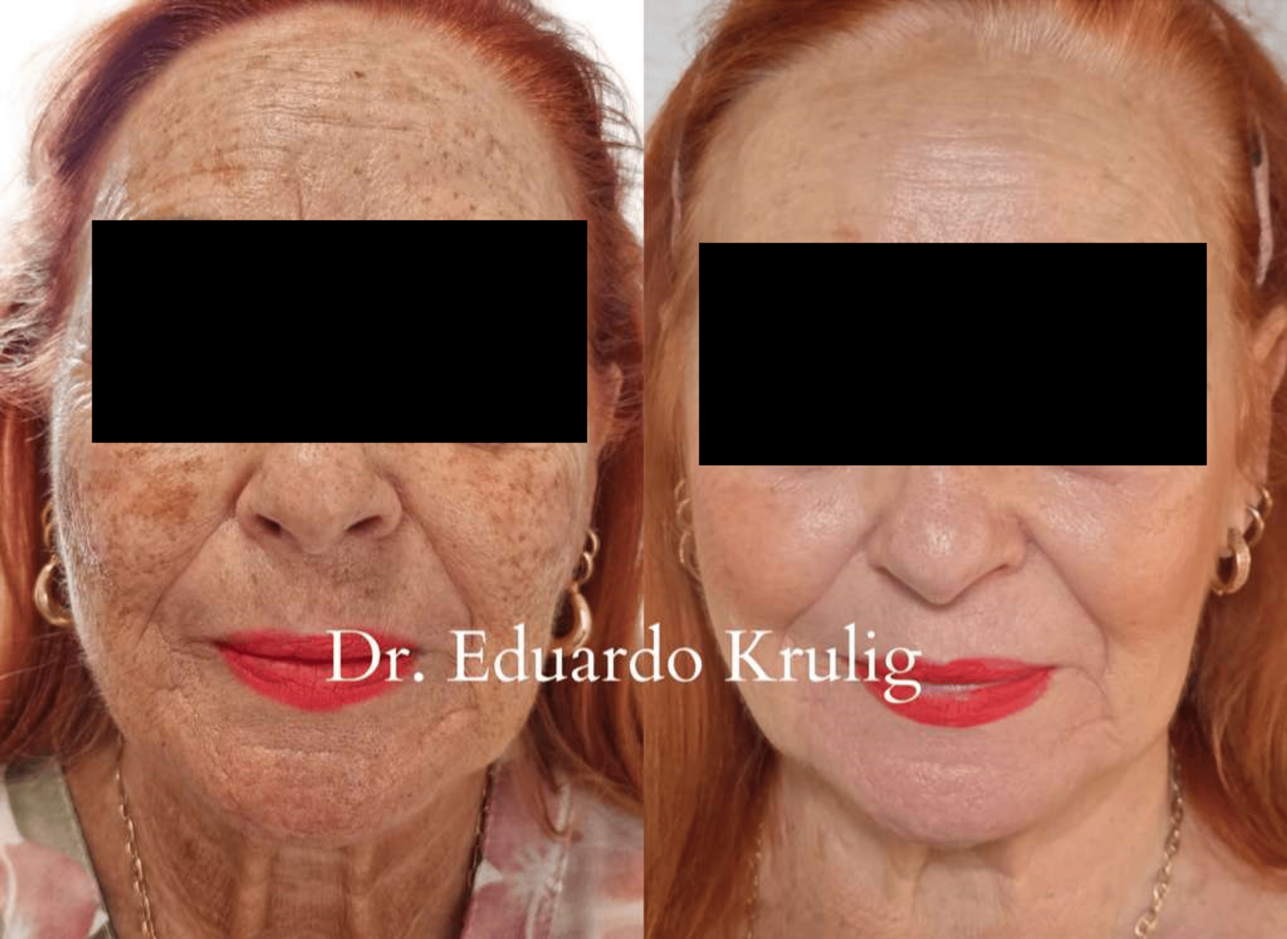
Before and after depigmentation of facial melasma and lentigines using the NewMelan protocol Clinical photographs of a 75-year-old female patient with diffuse facial melasma and lentigines. Left: baseline presentation showing extensive hyperpigmentation, uneven skin tone, and lentiginous spots. Right: two weeks after treatment with the NewMelan multimodal depigmentation protocol, demonstrating visible reduction in pigmentation, improved skin uniformity, and subtle enhancement of skin texture. Written informed consent was obtained for publication of these images

**Figure 2:**
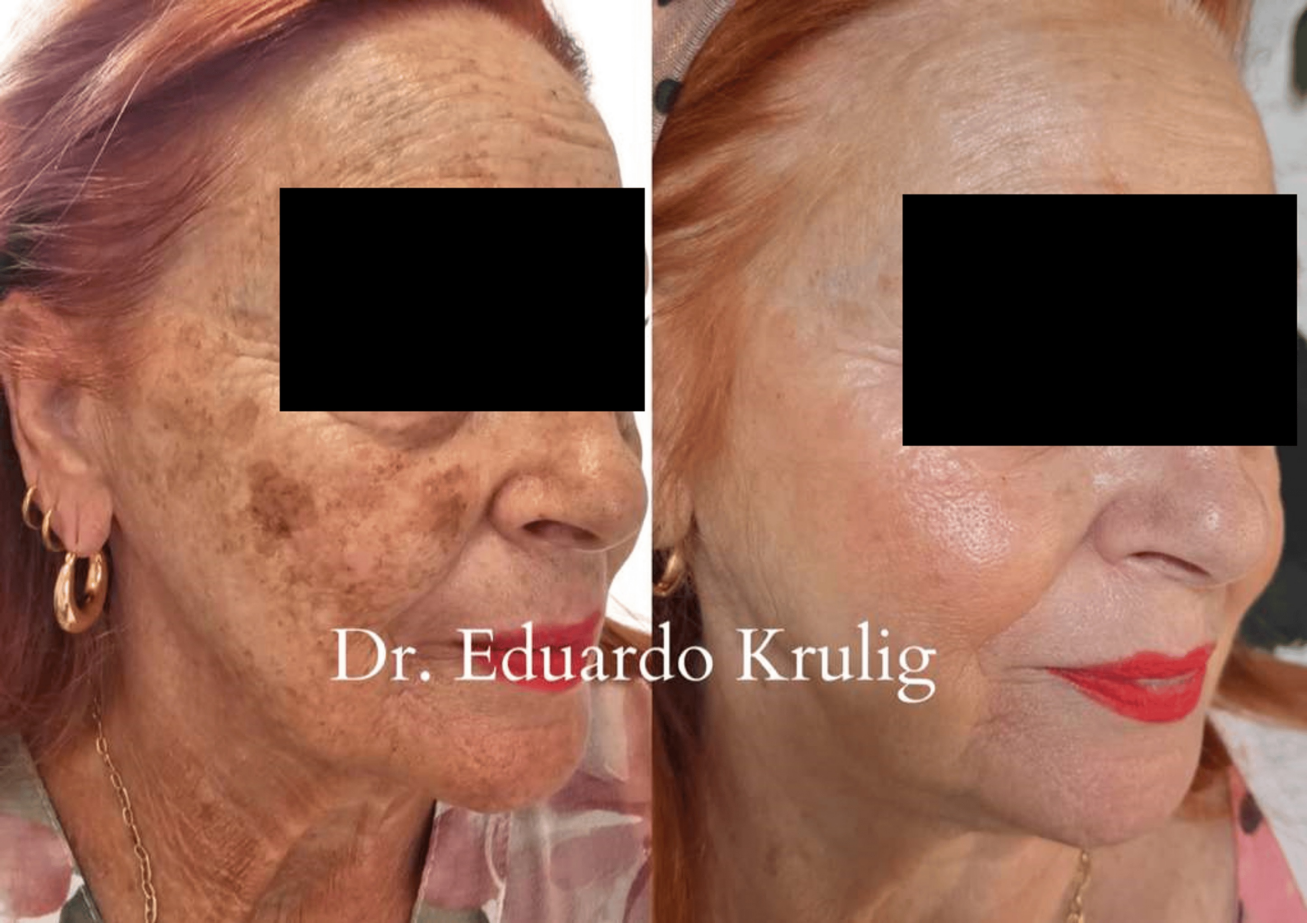
Before and after depigmentation of facial melasma and lentigines using the NewMelan protocol Clinical photographs of a female patient with diffuse facial melasma and lentigines. Left: baseline presentation showing prominent hyperpigmentation, uneven skin tone, and lentiginous spots on the malar region. Right: two weeks after treatment with the NewMelan multimodal depigmentation protocol, showing visible reduction in pigmentation, improved skin uniformity, and subtle enhancement of skin texture. The patient's eyes have been obscured for privacy. Written informed consent was obtained for publication of these images

**Figure 3:**
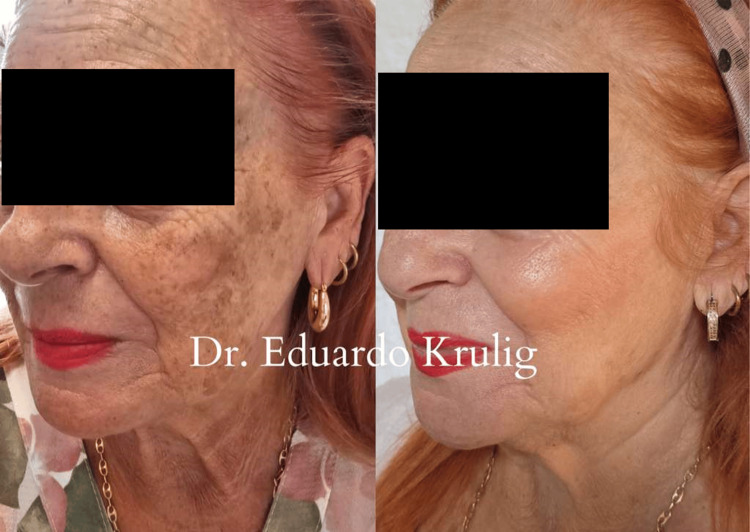
Before and after depigmentation of facial melasma and lentigines using the NewMelan protocol Clinical photographs of a female patient with diffuse facial melasma and lentigines. Left: baseline presentation showing prominent hyperpigmentation, uneven skin tone, and lentiginous spots on the malar region and cheek. Right: two weeks after treatment with the NewMelan multimodal depigmentation protocol, showing visible reduction in pigmentation, improved skin uniformity, and subtle enhancement of skin texture. The patient's eyes have been obscured for privacy. Written informed consent was obtained for publication of these images

Marked clinical improvement was documented after approximately two weeks, with substantial reduction in pigmentation intensity and improved skin tone uniformity. Serial clinical photographs demonstrated visible reduction in melasma and lentigines without adverse effects (Figures 1-3). The treated areas appeared more even, and skin texture was observed to improve subtly, likely due to enhanced epidermal turnover.

The patient was instructed to continue daily maintenance therapy. No complications or adverse events were reported during the treatment period. This case highlights the potential short-term efficacy of the NewMelan multimodal depigmentation protocol in improving facial hyperpigmentation in patients with melasma and lentigines.

## Discussion

Diffuse melasma and lentigines are complex pigmentary disorders that involve increased melanin production and irregular melanosome distribution [1]. The process of melanogenesis depends on multiple enzymatic pathways, particularly the activity of tyrosinase, which catalyzes the conversion of tyrosine to melanin [2]. Modern depigmentation strategies include agents that modulate tyrosinase activity, inhibit melanin synthesis, and enhance epidermal renewal, thereby reducing accumulated pigment [3].

The NewMelan multimodal protocol used in this case integrates these principles through a topical formulation containing arbutin 1.5%, kojic acid 1.0%, and azelaic acid 1.5%, administered according to a structured application schedule designed to target melanogenic pathways and accelerate epidermal turnover [4]. Arbutin inhibits tyrosinase activity, kojic acid suppresses melanogenesis by chelating copper at the active site of tyrosinase, and azelaic acid selectively inhibits abnormal melanocyte activity while also providing anti-inflammatory effects [5,6]. By targeting multiple stages of pigment formation and distribution, the protocol seeks to reduce melanin accumulation while promoting a more uniform skin tone [6]. Its depigmenting effects are achieved without apparent damage to melanocytes, allowing continued use as part of a maintenance strategy intended to minimize recurrence [7]. In addition to pigment reduction, improvement in skin texture, brightness, and overall facial appearance was observed in the present case, contributing to a subtle rejuvenating and skin-tightening effect [8].

The observed reduction in melasma and lentiginous pigmentation in this patient may reflect these underlying biological effects [9]. Marked clinical improvement was documented within approximately two weeks of treatment initiation, with substantial reduction in pigmentation intensity and improvement in overall skin appearance [10]. Serial clinical photographs demonstrated visible improvement without adverse effects [11].

The patient continued maintenance therapy following the initial treatment phase. No adverse effects or treatment-related complications were observed during the follow-up period [12-14].

An additional practical aspect of the NewMelan protocol is its relatively straightforward application schedule and noninvasive nature. These characteristics may facilitate its incorporation into routine clinical practice for the management of melasma, lentigines, and related pigmentary disorders. In the present case, the treatment was well tolerated and associated with noticeable clinical improvement, supporting further investigation of its potential role in dermatologic and aesthetic medicine.

Further research is needed to formally evaluate the efficacy, mechanisms, and long-term outcomes of structured multimodal depigmentation regimens, including prospective studies with standardized objective measures, larger patient populations, and longer follow-up periods.

## Conclusions

This case demonstrates short-term clinical improvement in diffuse melasma and lentigines using the NewMelan multimodal depigmentation protocol. The treatment may reduce hyperpigmentation through modulation of melanogenesis, inhibition of tyrosinase activity, reduction of oxidative processes, and interference with melanin transfer, leading to improved skin tone uniformity and substantial pigment reduction. In the present case, marked improvement in pigmentation, skin texture, brightness, and overall facial appearance was observed within approximately two weeks of treatment initiation. Continued maintenance therapy may help sustain clinical improvement and minimize recurrence over time. Further studies with larger patient populations and longer follow-up periods are necessary to evaluate the long-term efficacy, recurrence rates, and broader applicability of the NewMelan multimodal depigmentation protocol.
